# Changes in ocular surface and Meibomian gland after penetrating Keratoplasty

**DOI:** 10.1186/s12886-021-01851-4

**Published:** 2021-02-15

**Authors:** Kang Yoon Kim, Byunghoon Chung, Eung Kweon Kim, Kyoung Yul Seo, Ikhyun Jun, Tae-im Kim

**Affiliations:** 1 Department of Ophthalmology, Institute of Vision Research, Yonsei University College of Medicine, Seoul, Republic of Korea; 2 Department of Ophthalmology, International St. Mary’s Hospital, Catholic Kwandong University College of Medicine, Incheon, Republic of Korea; 3 Saevit Eye Hospital, Goyang, Republic of Korea; 4 Corneal Dystrophy Research Institute, Department of Ophthalmology, Yonsei University, College of Medicine, Seoul, Republic of Korea

**Keywords:** Penetrating keratoplasty, Meibomian gland dysfunction, Dry eye disease

## Abstract

**Background:**

To acquire desirable outcomes of penetrating keratoplasty (PKP), various factors affecting graft survival, visual function, and subjective symptom should be considered. As ocular surface and meibomian gland function are associated with these factors, this study aims to investigate changes of ocular surface and meibomian gland parameters after PKP.

**Methods:**

This retrospective case series study included 24 eyes of 24 patients who underwent penetrating keratoplasty. Examinations on lipid layer thickness (LLT), meiboscore, tear meniscus area (TMA), tear breakup time (TBUT), corneal fluorescein staining (CFS), Schirmer I test (SIT), Ocular Surface Disease Index (OSDI), and meibomian gland functions were performed before and at 1 week, 1 month, 6 months, and 12 months after surgery.

**Results:**

Compared to baseline (2.9 ± 0.6 s), TBUTs were longer at 1 week (4.4 ± 0.5 s, *P* = 0.027) and 6 months (4.4 ± 0.5, *P* = 0.048) after surgery. CFS values improved from baseline (6.5 ± 1.1) to 6 months (3.5 ± 0.6, *P* = 0.023) and 12 months (3.3 ± 0.7, *P* = 0.001) after surgery. Meibum quality value worsened at 1 week and 12 months after surgery and meibomian gland expressibility value worsened at 1 week and 6 months after surgery compared to baseline. OSDI scores improved at 6 and 12 months after surgery. Meiboscore showed no change throughout the follow up period. The patients with high preoperative meiboscore had worse meibomian gland expressibility at 6 and 12 months and meibum quality at 6 months postoperatively compared to their baseline and to those of patients with low preoperative meiboscore.

**Conclusions:**

After penetrating keratoplasty, ocular surface parameters including corneal staining, TBUT, and OSDI significantly improved whereas meibomian gland parameters showed deteriorations, which was marked in patients with high preoperative meiboscore. Thus, perioperative management of MGD is recommended for patients who undergo penetrating keratoplasty, especially in patients with advanced MGD.

## Background

Penetrating keratoplasty (PKP) is still a widely performed corneal transplantation procedure for corneal perforation, full thickness corneal opacity, corneal dystrophies, bullous keratopathy, and advanced keratoconus, when the diseased condition is difficult to treat with medications such as ointments, bandage contact lenses, autologous serum, platelet-rich plasma, or lamellar keratoplasty [1–4]. When seeking satisfying outcomes of PKP, it is important to consider various factors that affect graft survival, visual function, and subjective symptoms [1–3]. Common causes for graft failure include allograft rejection, endothelial decompensation, and ocular surface diseases (infectious or sterile keratitis, corneal scarring, etc.) [5]. Additional risk factors for graft failure are preoperative diagnosis, history of ocular infection/inflammation, and ocular surface complications during follow-up (epithelial defect, blepharitis) [6]. Delayed epithelial healing or persistent epithelial defect after keratoplasty, which aggravates infection, melting, scarring, and neovascularization, may restrict graft survival [5, 7]. Even though ocular surface abnormality has been underrated among the factors related to successful clinical outcome after PKP, it not only plays an important role in visual function and subjective symptoms, but is also a vital factor in graft survival [5, 8]. Ocular surface abnormalities with positive corneal staining are common after PKP, and tear film instability has been reported to influence visual function postoperatively [8]. Unstable tear film from the neurotrophic state, aqueous tear deficient state, and persistent ocular surface inflammation after keratoplasty cause dry eye disease (DED), persistent epithelial defect, neovascularization, and even allograft rejection [9].

In keratoplasty, tear film and ocular surface are disrupted by various insults. Keratoplasty severs the corneal nerve and leads to a neurotrophic state, which changes tear secretion, eyelid blinking, and epithelial growth [9, 10]. Changes in ocular surface anatomy affect normal tear film distribution [11]; subsequent tear film instability and dry eye disease in turn damage visual function and ocular surface integrity [12]. In this context, maintaining corneal epithelium, tear film function, and meibomian gland function to achieve homeostasis of the ocular surface is linked to the postoperative outcome of keratoplasty [12, 13].

Previous reports have studied changes of tear function, fluorescein corneal staining, and corneal sensitivity to assess ocular surface abnormalities after keratoplasty [8, 14]. However, despite the ever-growing attention on the role of meibomian gland function in ocular surface homeostasis, ocular surface and meibomian gland dysfunction (MGD) after keratoplasty has not yet been comprehensively studied [13]. In this study, we investigated dry eye disease after keratoplasty by evaluating ocular surface and meibomian gland parameters.

## Methods

This retrospective case series study was approved by the Severance Hospital Institutional Review Board, Seoul, South Korea (No. 1–2016-0027) and followed the tenets of the Declaration of Helsinki.

### Subjects

Twenty-four eyes from 24 patients who received PKP between January 2017 and December 2018 were enrolled in the study. All the patients were followed up for at least 12 months. Exclusion criteria included a history of previous keratoplasty, limbal deficiency, Sjogren’s syndrome, uncontrolled intraocular pressure, and rejection episode after the surgery. This study included 24 eyes of 24 patients with a mean age of 54.54 ± 14.39 (range, 31–82 years). Indications for keratoplasty were bullous keratopathy (10 eyes), corneal opacity (9 eyes), and advanced keratoconus (5 eyes).

### Surgical technique

PKP was performed by a single experienced surgeon (T.I.K.) following standardized procedures under general anesthesia. The recipient cornea trephination diameter was 7 to 8 mm and the donor corneal diameter was the same or 0.25 mm larger than that of the recipient’s. The graft was sutured to the recipient bed with 16 interrupted 10–0 nylon sutures. At the end of the surgery, intraoperative adjustment of sutures was performed to minimize the risk of corneal astigmatism. Pressure patch was applied for 3 to 5 days until epithelial defects are healed.

Postoperatively, topical 0.5% levofloxacin (Cravit, Santen Pharmaceutical, Osaka, Japan) and topical 1% prednisolone acetate (PredForte, Allergan, Irvine, California) were applied four times a day. Topical antibiotics were gradually tapered and maintained for 1 year. Topical steroids were also tapered and maintained for 2 years. Increased intraocular pressure was managed with glaucoma medication and topical 1% prednisolone was switched to topical 0.5% loteprednol (Lotemax, Bausch & Lomb, Rochester, New York) in each patient as needed. Patients were instructed to use 0.1% hyaluronic acid artificial tears for dry eye management, as necessary.

### Outcome measures

Detailed examinations of the ocular surface and meibomian gland function were performed by one of the authors (IJ) before surgery and at 1 week, 1 month, 6 months, and 12 months after surgery.

LLT and noninvasive meibography were obtained from LipiView II interferometer (TearScience, Morrisville, NC). Meiboscore was measured from grade 0 (no loss of gland) to grade 3 (loss of more than two-thirds of the total gland area) as previously described [15]. TMA of the lower eyelid was measured using Fourier-domain optical coherence tomography (OCT; RTVue; Optovue, Inc., Fremont, CA). Vertical 2-mm scan images at the middle of the lower eyelid were obtained twice for each eye and TMA was calculated using ReVue software (version 4.0; Optovue, Inc., Fremont, CA) [16]. TBUT was measured three times using a fluorescein strip (Haag-Streit, Koeniz, Switzerland), and the mean value was collected. Subsequent CFS was graded from 0 to 15 according to the National Eye Institute (NEI) scale [17]. SIT without anesthesia was performed for 5 min at least 10 min after corneal staining. Subjective symptoms were assessed using the OSDI questionnaire [18]. Meibomian gland functions were assessed in terms of lid margin abnormality, gland expressibility, and meibum quality. Lid margin abnormalities were graded from 0 to 4 based on the presence of vascular engorgement, plugged Meibomian gland orifices, displacement of the mucocutaneous junction, and irregularity of the lid margin [19]. Meibomian gland expressibility was checked by applying firm digital pressure on the central five glands of the lower eyelid and was measured as grade 0 if all five glands expressed and grade 3 if none of the glands expressed [20]. Meibum quality of eight lower lid glands was graded from 0 (clear) to 3 (toothpaste-like) and a total score of up to 24 was acquired [20].

### Statistical analysis

All data were analyzed using SPSS for Windows version 23.0 (SPSS Inc., Chicago, IL) and expressed as mean ± standard deviation. Repeated measure analysis of variance (RM-ANOVA) was used to analyze the differences between visits. Paired *t-*test and Wilcoxon signed-rank test were used to compare data between two time points within a group, with adjustment of the level of significance according to the Bonferroni correction. Subgroup analysis and comparison with different characteristics were performed using independent samples *t*-test and Mann-Whitney test. *P* values less than 0.05 were considered statistically significant.

## Results

### Ocular surface and Meibomian gland status after Keratoplasty

**Fig. 1:**
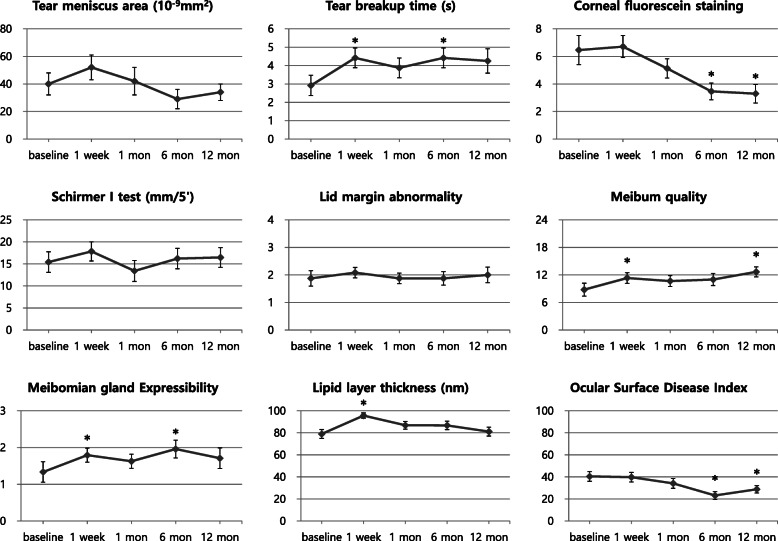
Clinical parameters of dry eye disease over time. *Statistical significance (*P* < 0.05) for changes between baseline and follow-up period

The ocular surface and meibomian gland parameters of DED after keratoplasty are shown in Table 1. TBUT values were higher at 1 week and 6 months after surgery than the baseline. Corneal staining scores showed a significant reduction from the baseline at 6 and 12 months after surgery. Meibum quality values were elevated at 1 week and 12 months after surgery. Meibomian gland expressibility values were elevated at 1 week and 6 months after surgery. Meiboscore showed no change throughout the follow-up period. LLT was higher at 1 week postoperatively than the baseline. OSDI scores were improved at 6 and 12 months after surgery compared to the baseline. Other clinical parameters of DED after surgery were not significantly different from the baseline. Figure 1 shows the changes in DED parameters over the course of keratoplasty.

**Table 1:** Clinical Parameters of Dry Eye Disease

	Baseline	1 week		1 month		6 months		12 months	
		Value	*P* (vs Baseline)	Value	*P* (vs Baseline)	Value	*P* (vs Baseline)	Value	*P* (vs Baseline)
Tear meniscus area (10^−9^ mm2)	40 ± 8	52 ± 9	0.179	42 ± 10	0.819	29 ± 7	0.290	34 ± 6	0.595
TBUT (sec)	2.9 ± 0.6	4.4 ± 0.5	0.027^a^	3.9 ± 0.5	0.259	4.4 ± 0.5	0.048^a^	4.3 ± 0.7	0.096
Corneal fluorescein staining score (0–15)	6.5 ± 1.1	6.7 ± 0.8	0.762	5.1 ± 0.7	0.283	3.5 ± 0.6	0.023^a^	3.3 ± 0.7	0.001^a^
Schirmer’s test I value (mm/5′)	15.4 ± 2.3	17.8 ± 2.2	0.356	13.4 ± 2.4	0.491	16.2 ± 2.3	0.764	16.5 ± 2.2	0.662
Lid margin abnormality (0–4)	1.9 ± 0.3	2.1 ± 0.2	0.410	1.9 ± 0.2	1.000	1.9 ± 0.2	1.000	2.0 ± 0.3	0.689
Meibum quality (0–24)	8.8 ± 1.4	11.3 ± 1.1	0.033^a^	10.7 ± 1.2	0.136	11.0 ± 1.3	0.161	12.7 ± 1.1	0.023^a^
Meibomian gland expressibility (0–3)	1.3 ± 0.2	1.8 ± 0.2	0.024^a^	1.6 ± 0.2	0.231	2.0 ± 0.2	0.019^a^	1.7 ± 0.2	0.083
Meiboscore (0–3)	1.2 ± 0.2	1.3 ± 0.2	–	1.2 ± 0.2	–	1.3 ± 0.2	–	1.3 ± 0.2	–
Lipid layer thickness (nm)	79.0 ± 4.1	95.6 ± 2.5	0.006^a^	86.9 ± 3.6	0.168	86.7 ± 3.8	0.158	81.1 ± 4.1	0.694
OSDI (0–100)	40.45 ± 4.42	39.73 ± 4.28	0.879	34.13 ± 4.42	0.070	23.14 ± 3.52	0.001^a^	28.78 ± 3.37	0.002^a^

*TBUT* tear break up time, *OSDI* ocular surface disease index

Results are expressed as mean ± SD

^a^Statistical significance (*P* < 0.05)

### Changes in parameters in groups with different Meiboscores

**Fig. 2:**
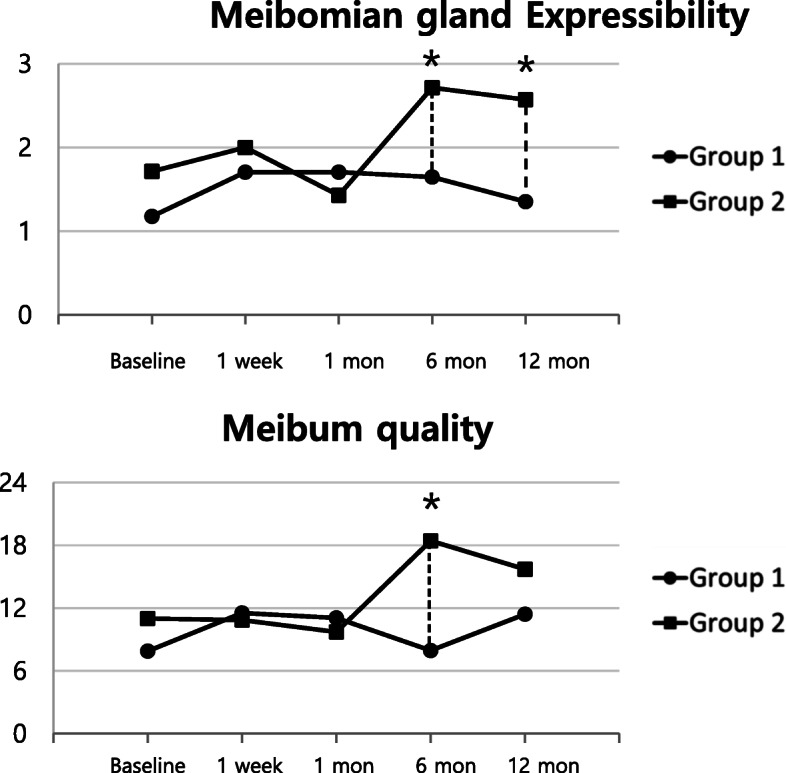
Clinical parameters of dry eye disease in groups with different meiboscores. Group 1, meiboscore 0–1; Group 2, meiboscore 2–3. *Statistical significance (*P* < 0.05) for changes between groups at each time point

Meiboscore of each patient remained unchanged from the baseline through the whole follow-up period. Patients were divided into two groups with different meiboscores; group 1, levels 0–1 and group 2, levels 2–3 from the baseline examination. Ocular surface parameters and meibomian gland parameters between the two groups at each time point were compared (Fig. 2). Compared to the baseline, the high meiboscore group showed significant worsening of meibomian gland expressibility values at 6 and 12 months and meibum quality values at 6 months after surgery. When compared to the low meiboscore group, meibomian gland expressibility values in the high meiboscore group were significantly worse at 6 and 12 months after surgery, and meibum quality values in the high meiboscore group were significantly worse at 6 months after surgery (Table 2). Other parameters showed no statistical differences between the groups at each time point.

**Table 2:** Meibomian Gland Parameters of Groups with Different Meiboscore

	Group 1		Group 2		***P***
	Value	*P* (vs Baseline)	Value	*P* (vs Baseline)	
**Meibomian gland expressibility (0–3)**					
Baseline	1.2 ± 0.9		1.7 ± 0.8		0.198
1 week	1.7 ± 0.9	0.046^a^	2.0 ± 1.0	0.356	0.494
1 month	1.7 ± 1.0	0.095	1.4 ± 0.8	0.356	0.536
6 months	1.7 ± 0.9	0.149	2.7 ± 0.5	0.038^a^	0.006^b^
12 months	1.4 ± 1.0	0.455	2.6 ± 1.1	0.048^a^	0.02^b^
**Meibum quality (0–24)**					
Baseline	7.9 ± 7.2		11.0 ± 6.2		0.325
1 week	11.5 ± 6.0	0.021^a^	10.9 ± 4.8	0.915	0.796
1 month	11.5 ± 6.0	0.034^a^	9.7 ± 5.3	0.580	0.611
6 months	7.9 ± 4.1	0.970	18.4 ± 5.0	0.045^a^	0.001^b^
12 months	11.4 ± 4.7	0.086	15.7 ± 5.8	0.168	0.07

Group 1, meiboscore 0–1; Group 2, meiboscore 2–3

Results are expressed as mean ± SD

^a^Statistical significance (*P <* 0.05) between baseline and follow up period in each group

^b^Statistical significance (*P* < 0.05) between two groups at each time point

### Effect of Antiglaucoma medication usage on parameters

The number of patients who used antiglaucoma medications for over 1 month was 11 and those who did not were 13. These two groups of patients were compared to analyze the effect of antiglaucoma medications on the entire ocular surface and meibomian gland parameters. SIT values were significantly higher in the group that used antiglaucoma medications in the pre- and postoperative period.

## Discussion

In this study, we analyzed changes in the ocular surface and meibomian gland parameters over 12 months of follow-up for PKP. We found that the corneal fluorescein score, TBUT, and OSDI showed a significant improvement over time. We also found that meibomian gland functions, such as meibomian gland expressibility and meibum quality, significantly deteriorated without structural changes after PKP, and that the extent of deterioration was more prominent in patients with preexisting MGD.

PKP has been a widely used corneal transplantation procedure for various corneal disorders. This procedure involves removal of the full-thickness cornea, total severance of the corneal nerve, intraocular manipulation, and extensive suturing with resulting ocular surface irregularity. Even after uneventful surgery, ocular discomfort occurs. A previous study reported that superficial punctate keratopathy and dry eye were common complications of corneal denervation after PKP [21]. Aggravation of DED parameters, such as TBUT, corneal fluorescein stain, Schirmer I test, and corneal esthesiometry, after keratoplasty, has been previously reported [11, 14, 22]. Distinct from previous reports, the current study investigated meibomian gland parameters in addition to ocular surface parameters as the two are not separable.

Corneal staining scores showed gradual improvement from 1 week after surgery to 12 months after surgery. However, some degree of epithelium damage persisted even after 12 months. The poor corneal staining scores at the baseline were caused by primary corneal diseases. As the preoperative diagnosis of cornea was treated by keratoplasty and harmful injuries on epithelium induced by pathologic corneas were cessated, the corneal epithelium regained its integrity and staining scores improved from baseline which is consistent with a previous report by Lin et al. [14] Corneal epithelium defect after keratoplasty gradually improved but did not completely heal until the last study visit. The remaining epithelial defect after keratoplasty are attributable to denervation, disconnection from limbal stem cells, frequent use of eye drops, and aggravation of DED [9, 22]. And deterioration of meibomian gland function which was observed in our study also causes epithelial damage via released inflammatory mediators and lipids on the ocular surface [23].

Compared to the measurement at the baseline, TBUTs significantly improved at 1 week and 12 months after surgery. Despite the fact that tear meniscus areas and Schirmer 1 test values showed no significant changes compared to those of baseline, the increase in TBUTs demonstrates an improvement in tear film stability without changes in tear production or volume. OSDI scores at 6 and 12 months after surgery were lower than those at the baseline. Surgical removal of pathologic cornea and subsequent corneal denervation after keratoplasty may have mitigated dry eye sensation at the early postoperative period. However, restoration of corneal sensation and deterioration of MGD led to increase in OSDI scores at 12 months after surgery.

Comprehensive assessment of meibomian gland parameters using slit-lamp microscopy, meibography, and interferometry was performed. Meibomian gland expressibility worsened at 1 week and 6 months after surgery. Meibum quality scores showed a trend of worsening after surgery, and the changes, compared to those at the baseline, were significant at 1 week and 12 months postoperatively. Lid margin abnormality score and meiboscore showed no statistically significant change during the follow-up period. MGD is caused by the stagnation of meibum inside the glands, dilation of the ductal system, and consequent loss of glands. Our hypothesis was that PKP may aggravate all aspects of MGD. However, only the functional parameters of meibomian glands, such as expressibility and meibum quality, were altered without any structural changes in the lid margin and meibomian gland tissues.

Causes of functional changes in meibomian glands are multifactorial, and the exact underlying mechanism is unclear. Damage in neural regulation of meibomian glands may affect meibum secretion. Meibomian glands, which are surrounded by a dense meshwork of cholinergic parasympathetic nerve fibers may have a role in the neural feedback loop [24, 25]. As the denervation of the cornea by keratoplasty is known to interfere with the feedback loop of the lacrimal functional unit [23], a possible neural dysregulation of meibomian glands after keratoplasty may contribute to meibum secretion abnormalities.

Insufficient lid hygiene after keratoplasty may contribute to the functional deterioration of the meibomian glands. Lid hygiene is known to reduce lipid by-products and lipolytic bacteria on the lid margin, which can reduce the level of ocular surface MMP-9, improve the quality of the lipid layer, and alleviate MGD [26–28]. After keratoplasty, patients are usually discouraged from cleaning their eyelids because of the risk of mechanical damage to the ocular surface. Lack of lid hygiene, which causes stagnation of lipids and obstruction of the gland, may cause functional changes of meibomian gland after surgery.

The effects of preservatives and antiglaucoma medications on meibomian glands have been previously reported, and are known to cause functional and structural changes in meibomian glands [29–31]. Antiglaucoma medications cause subclinical inflammation which results in keratinization of meibomian gland orifice and subsequent stagnation of meibum [29]. Lee et al. demonstrated worsening of lid margin abnormality, expressibility of meibum, and meiboscore in patients using preservative-containing antiglaucoma medications [30]. However, in our study, the groups with or without antiglaucoma medications only differed in SIT values. Because our study was unable to control the variables related to antiglaucoma medication usage, the results could not reflect the impact of antiglaucoma medication on the ocular surface and meibomian glands. Taken together, the mechanism of MGD after keratoplasty may involve various insults, including damaged neural regulation of meibomian glands, insufficient lid hygiene, preservative-containing eyedrops, and antiglaucoma medications.

Functional changes of meibomian glands after surgery have been previously reported in surgeries other than PKP [32–36]. Unavoidable trauma from cataract surgery may induce ocular inflammation [37]. Toxicity from eye drops [38] and lid dysfunction because of intraoperative use of lid speculum may also contribute to MGD. Our results are similar to those reported for cataract surgeries in that no structural changes occurred while meibomian gland function deteriorated. It can be speculated that cataract surgery interferes with the ocular surface and meibomian glands in ways similar to PKP, but their exact mechanism could not be elucidated in the current study.

At the baseline, patients were divided into two groups; high and low meiboscore groups, and the parameters between the two groups were compared. Functional parameters, meibomian gland expressibility, and meibum quality score showed statistically significant differences between the two groups. High meiboscore group had worse expressibility and meibum quality at 6 and 12 months after surgery. Patients with worse preexisting MGD were more prone to the functional deterioration of meibomian glands without any change in meiboscore. MGD starts with the stagnation of meibomian gland secretion, and chronic inflammation within the glands leads to subsequent structural changes. Therefore, patients with structural damage of the glands are more likely to have long-standing MGD and frail gland function, which could easily be affected by harmful insults that lead to functional degradation of the glands.

As a limitation of our study, the sample size was relatively small due to infrequent cases of keratoplasty. Moreover, a control group was lacking. In addition, there could be numerous variables affecting the DED parameters that were not addressed in our study, which can confound the results. Furthermore, different use of eye drops, such as steroids, antiglaucoma medications, and artificial tears, among study participants could not be controlled and could have affected the results. Increased intraocular pressure after keratoplasty is unpredictable and the response to treatment is not uniform. Thus, use of antiglaucoma medication after surgery is individualized. The number of antiglaucoma medications and duration of use are too heterogeneous in patients and very complicated to control. Further studies with meticulous study design are needed to assess the effect of the antiglaucoma medication in post-keratoplasty patients.

In spite of these limitations, our study showed significant clinical improvement in ocular surface conditions, including better corneal staining and OSDI, and elongation of TBUT. Although greater deterioration of the functional parameters of MGD after PKP was observed for the entire observation period, especially among the patients with an advanced stage of MGD, our findings suggest that thorough observation of MGD before and after keratoplasty is necessary to identify and manage ocular surface and meibomian gland deficits to achieve more desirable post-keratoplasty results.

## Data Availability

The datasets generated analyzed during the current study are not publicly available due to patient’s data privacy but can be made available from the corresponding author upon reasonable request.

## Abbreviations

PKP: Penetrating keratoplasty
LLT: Lipid layer thickness
TMA: Tear meniscus area
TBUT: Tear breakup time
CFS: Corneal fluorescein staining
SIT: Schirmer I test
OSDI: Ocular Surface Disease Index
DED: Dry eye disease
MGD: Meibomian gland dysfunction
