# Correction to: Changes in ocular surface and Meibomian gland after penetrating Keratoplasty

**DOI:** 10.1186/s12886-021-01890-x

**Published:** 2021-03-05

**Authors:** Kang Yoon Kim, Byunghoon Chung, Eung Kweon Kim, Kyoung Yul Seo, Ikhyun Jun, Tae-im Kim

**Affiliations:** 1 Department of Ophthalmology, Institute of Vision Research, Yonsei University College of Medicine, Seoul, Republic of Korea; 2 Department of Ophthalmology, International St. Mary’s Hospital, Catholic Kwandong University College of Medicine, Incheon, Republic of Korea; 3 Saevit Eye Hospital, Goyang, Republic of Korea; 4 Department of Ophthalmology, Corneal Dystrophy Research Institute, Yonsei University, College of Medicine, Seoul, Republic of Korea

**Correction to: BMC Ophthalmol 21, 85 (2021)**

**https://doi.org/10.1186/s12886-021-01851-4**

Following publication of the original article [1], we were notified that Ikhyun Jun should have also been marked as a corresponding author.

The original article has been corrected.
